# Sphingosine-1-phosphate levels are inversely associated with left ventricular and atrial chamber volume and cardiac mass in men: The Study of Health in Pomerania (SHIP)

**DOI:** 10.1007/s00392-023-02200-9

**Published:** 2023-04-25

**Authors:** Jan Schielke, Till Ittermann, Stefan Groß, Eileen Moritz, Matthias Nauck, Nele Friedrich, Edzard Schwedhelm, Bernhard H. Rauch, Henry Völzke, Robin Bülow, Bishwas Chamling, Stephan Burkhard Felix, Martin Bahls, Marcus Dörr, Marcello Ricardo Paulista Markus

**Affiliations:** 1 Department of Internal Medicine B, Cardiology, Angiology, Pneumology and Internal Intensive Care Medicine, University Medicine Greifswald, Ferdinand-Sauerbruch-Straße, 17475 Greifswald, Germany; 2 German Centre for Cardiovascular Research (DZHK), Partner Site Greifswald, Greifswald, Germany; 3 Department of Study of Health in Pomerania/Clinical-Epidemiological Research, Institute for Community Medicine, University Medicine Greifswald, Greifswald, Germany; 4 Department of General Pharmacology, Institute of Pharmacology, University Medicine Greifswald, Greifswald, Germany; 5 Institute of Clinical Chemistry and Laboratory Medicine, University Medicine Greifswald, Greifswald, Germany; 6 Institute of Clinical Pharmacology and Toxicology, University Medical Center Hamburg-Eppendorf, Hamburg, Germany; 7 German Centre for Cardiovascular Research (DZHK), Partnerartner Site Hamburg/Kiel/Lübeck, Hamburg, Germany; 8 Department of Human Medicine, Section of Pharmacology and Toxicology, Carl Von Ossietzky University of Oldenburg, Oldenburg, Germany; 9 Institute of Diagnostic Radiology and Neuroradiology, University Medicine Greifswald, Greifswald, Germany; 10 Division of Cardiovascular Imaging, Department of Cardiology I, University Hospital Münster, Münster, Germany; 11 German Center for Diabetes Research (DZD) Partner Site Greifswald, Greifswald, Germany

**Keywords:** Left ventricular geometry and function, Left ventricular mass, Left ventricular hypertrophy, Sphingosine-1-phosphate

## Abstract

**Aims:**

Sphingosine-1-phosphate (S1P) is a signaling lipid, which is involved in several cellular processes including cell growth, proliferation, migration and apoptosis. The associations of serum S1P levels with cardiac geometry and function are still not clear. We investigated the associations of S1P with cardiac structure and systolic function in a population-based sample.

**Methods and results:**

We performed cross-sectional analyses of 858 subjects (467 men; 54.4%), aged 22 to 81 years, from a sub-sample of the population-based Study of Health in Pomerania (SHIP-TREND-0). We analyzed the associations of serum S1P with structural and systolic function left ventricular (LV) and left atrial (LA) parameters as determined by magnetic resonance imaging (MRI) using sex-stratified multivariable-adjusted linear regression models. In men, MRI data showed that a 1 µmol/L lower S1P concentration was associated with an 18.1 mL (95% confidence interval [CI] 3.66–32.6; *p* = 0.014) larger LV end-diastolic volume (LVEDV), a 0.46 mm (95% CI 0.04–0.89; *p* = 0.034) greater LV wall thickness (LVWT) and a 16.3 g (95% CI 6.55–26.1; *p* = 0.001) higher LV mass (LVM). S1P was also associated with a 13.3 mL/beat (95% CI 4.49–22.1; *p* = 0.003) greater LV stroke volume (LVSV), an 18.7 cJ (95% CI 6.43–30.9; *p* = 0.003) greater LV stroke work (LVSW) and a 12.6 mL (95% CI 1.03–24.3; *p* = 0.033) larger LA end-diastolic volume (LAEDV). We did not find any significant associations in women.

**Conclusions:**

In this population-based sample, lower levels of S1P were associated with higher LV wall thickness and mass, larger LV and LA chamber sizes and greater stroke volume and work of the LV in men, but not in women. Our results indicate that lower levels of S1P were associated with parameters related with cardiac geometry and systolic function in men, but not in women.

**Graphical abstract:**

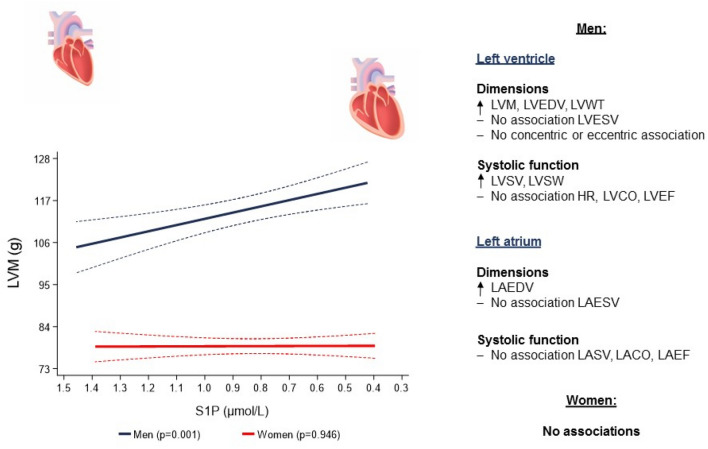

## Introduction

The last decades brought significant advances in the therapy of cardiovascular diseases (CVDs) and in particular in the field of heart failure. On the other hand, the prevalence of cardiovascular (CV) morbidity and its consequent health loss burden is still increasing, which is attributable in particular to an aging population [1, 2]. CVDs remain the most common reason of morbidity and mortality in Europe, USA and Asia with a significant economic impact on health and social security systems [1, 2].

The ability of the heart muscle to adapt to either pathological or physiological conditions is known as cardiac plasticity [3]. In a pathophysiological perspective, adverse remodeling of the heart is characterized by modification of cardiac shape, size, structure and function [4]. Sphingosine-1-phosphate (S1P) seems to possess physiologic functions that might influence cardiac remodeling.

S1P is a bioactive sphingolipid, transducing its endocrine effects through a group of G-protein coupled cell surface sphingosine-1-phosphate receptors (S1PR 1–5) [5, 6] and is involved in essential cellular processes including cell growth, proliferation, migration and apoptosis [7]. The role of S1P in the field of CVDs is becoming increasingly investigated [8–16]. Previous studies showed, that lower S1P concentrations were associated with deleterious cardiovascular outcomes [12, 14]. Furthermore, S1P mediates protective mechanisms against ischemic and reperfusion injury in cardiomyocytes [8–10, 13, 17, 18], enhances myocardial regeneration after myocardial infarction [11, 19] and is even attributed to be partially responsible for the advantageous effects of ß-blockers in patients with heart failure and cardiac remodeling after myocardial infarction [16, 20]. Altogether, these previous findings support the hypothesis that alterations of serum S1P levels, mainly lower levels, might be related to deleterious cardiovascular outcomes.

To the best of our knowledge, no previous population-based study has investigated the association of S1P concentrations with heart geometry and function. Therefore, the aim of the present study was to investigate the relation between lower S1P and LV and LA parameters of structure and systolic function as assessed by magnetic resonance imaging (MRI) in a large population-based sample.

## Materials and methods

### Study population

The present cross-sectional analysis is based on data from the population-based Study of Health in Pomerania (SHIP). The study design and recruitment strategy have been described elsewhere in detail [21]. Our analyses were based on data obtained from a sub-sample of the second SHIP cohort (SHIP-TREND-0) established between 2008 and 2012 [22]. In brief, a stratified random sample of 8,826 adults, aged 20–79 years, was selected from the population of West Pomerania, the north-eastern region of Germany. Participation in the first SHIP-START cohort was an exclusion criterion. In total 4,420 subjects participated in SHIP-TREND-0 (response 50.1%). Among them, 957 subjects (427 women, 44.6%), aged 21 to 81 years, who were eligible and willing to undergo whole-body MRI participated in the cardiac MRI substudy (Supplementary Figure I).

We excluded participants with previous self-reported myocardial infarction or stroke (*n* = 17), pacemaker (*n* = 1), left bundle block (*n* = 1) and a LVEF lower than 40% as determined by MRI (*n* = 9). We also excluded participants with missing values for S1P (*n* = 61) or any of the covariables used in the regression models (*n* = 10) (Supplementary Figure I). The final analytical sample comprised 858 subjects (391 women; 45.6%), aged 22–81 years (individuals with good quality images for the LV, *n* = 838 and for the LA, *n* = 775) (Supplementary Figure I).

All study participants gave written informed consent. The study was approved by the ethics committee of the University of Greifswald [22] and complies with the Declaration of Helsinki.

### Cardiac MRI

Cardiac MRI was performed on a 1.5-T MR system (Magnetom Avanto; Siemens Medical Systems, Erlangen, Germany) [23] with subjects in a supine position.

LV analysis was performed according to the post-processing guidelines of the Society for Cardiovascular Magnetic Resonance [24]. LV concentricity (LVC) was calculated as left ventricular mass (LVM)/left ventricular end-diastolic volume (LVEDV). Left ventricular stroke volume (LVSV), left ventricular stroke work (LVSW) [25], left ventricular cardiac output (LVCO) and left ventricular ejection fraction (LVEF) were calculated following the formulas described in the supplemental material.

For LA analysis, contours of end-diastolic and end-systolic endocardial borders were marked in transversal-axis in all phases. LA stroke volume (LASV), LA cardiac output (LACO) and LA ejection fraction (LAEF) were calculated following the formulas described in the supplemental material.

### Serum sphingosine-1-phosphate

Blood samples were taken from the cubital vein and analyzed directly or stored at − 80 °C in the Integrated Research Biobank of the University Medicine Greifswald [26]. Serum S1P was quantified by liquid chromatography tandem mass spectrometry (LC–MS/MS) with minor modifications as previously described [12]. After addition of 20 μL of the internal standard (1 μmol/L S1P-*d*_7_ [Avanti Polar Lipids, Alabaster, AL, USA]) to 20 μL serum, proteins were precipitated and passed to centrifugation. The sample isolates were subjected to reverse-phase chromatography and positive electrospray ionization. After elution with a binary gradient S1P was quantified by MS/MS in the multiple reaction mode, monitoring the (M + H) S1P parent ion (*m/z* = 380) fragmentation to the daughter ion (*m/z* = 264). The internal standard S1P-*d*_7_ with the m/z 387 to 271 transition was used to correct for variations in sample preparation and instrument response. Calibration curves were generated to calculate absolute S1P concentrations in the serum sample and quality controls were included and accepted with a coefficient of variation below 15% [12, 27].

### Interview, medical and laboratory examination

Information on age, sex, socio-economic variables and smoking status [28] was collected by trained and certificated medical staff during a standardized computer-assisted interview.

All participants underwent an extensive standardized medical examination, including anthropometric measurements and bioelectrical impedance analysis. Blood pressure was measured after a resting period of at least five minutes. Systolic and diastolic blood pressures as well as heart rate were measured three times on the right arm of seated subjects using an oscillometric digital blood pressure monitor (HEM-705CP, Omron Corporation, Tokyo, Japan) with an interval of three minutes between readings. The mean of the second and third measurements for the systolic and diastolic blood pressures and for the heart rate was calculated and used for the present analyses. Hypertension was defined as systolic blood pressure ≥ 140 mm Hg and/or diastolic blood pressure ≥ 90 mm Hg and/or current self-reported use of any anti-hypertensive medication.

Additionally, fasting and non-fasting blood samples were obtained from all study participants [29], to determine glycated hemoglobin, glucose concentrations, total serum cholesterol, low and high density lipoprotein-cholesterol (LDL-C, HDL-C), serum creatinine and estimated glomerular filtration rate (eGFR).

### Statistical analysis

To characterize the study sample, data was reported as the median (25th and 75th percentile) for continuous variables and as percentages for categorical variables stratified by tertiles of S1P and sex.

While the association of S1P concentrations with MRI determined LVM was not modified by age (*p*-value for interactio*n* = 0.369), it was modified by sex (*p*-value for interactio*n* = 0.013). Consequently, we decided to evaluate all the associations of S1P with LV and LA parameters stratified by sex and adjusted for age, body fat mass, body fat-free mass, height^2.7^, systolic blood pressure, use of antihypertensive medication, glycated hemoglobin, use of hypoglycemic medication, smoking status and estimated glomerular filtration rate (eGFR, calculated by the Chronic Kidney Disease-Epidemiology Collaboration [CKD-EPI] equation[30]). In order to evaluate the robustness of our findings in light of individuals that did not take part in the MRI examination, we performed inverse probability weighting[31], assuming a missing at random mechanism [32]. The inverse probability weights were calculated in logistic regression models with participation in the MRI examination as outcome and sociodemographic and health-related variables as predictors. We used fractional polynomials to test potential non-linear relationships between S1P levels and the outcome variables [33].

In sensitivity analyses, we explored the associations of S1P with LVM stratified by hypertension (yes/no) and by smoking status (never, former or current smoker). Hypertension was defined as systolic blood pressure ≥ 140 mmHg and/or diastolic blood pressure ≥ 90 mmHg and/or current self-reported use of any anti-hypertensive medication (Anatomical Therapeutic Chemical code C02, C03, C07, C08 and C09).

A two-sided *p*-value *p* < 0.05 was considered as statistically significant. Statistical analyses were performed using Stata 17.0 (Stata Corporation, College Station, TX, USA).

## Results

Table 1 shows descriptive data of the study participants stratified by sex-specific tertiles of serum S1P concentrations. The median age was similar between the tertiles for men and women. While the use of hypertensive medication was higher in the first tertile than in the other groups for men, it was similar in all groups in women. The levels of total cholesterol, LDL-cholesterol and HDL-cholesterol were lower in the first tertile than in the other groups for men, but the use of lipid-lowering medication was also higher in this group, when compared to the others. In women the use of lipid-lowering medication was higher in the first tertile group than the others, but there was no significant difference regarding the cholesterol levels. Men also showed a lower eGFR in the first tertile than the others groups. All other characteristics did not differ relevantly between the groups for men and women.

**Table 1:** Characteristics of the study sample stratified by tertiles of serum sphingosine-1-phosphate (S1P) levels and sex (*n* = 858)

Parameter		First tertile	Second tertile	Third tertile	Total	*p*-value*
*N* (%)	Men	156 (54.4)	156 (54.6)	155 (54.4)	467 (54.4)	
	Women	131 (45.6)	130 (45.5)	130 (45.6)	391 (45.6)	
Sphingosine-1-phosphate (µM)	Men	0.65 (0.60, 0.69)	0.81 (0.77, 0.84)	0.97 (0.93, 1.06)	0.81 (0.69, 0.93)	
	Women	0.66 (0.60, 0.70)	0.82 (0.79, 0.85)	1.00 (0.93, 1.09)	0.82 (0.70, 0.93)	
Age (years)	Men	52 (39, 65)	48 (40, 59)	47 (40, 49)	49 (40, 60)	0.145
	Women	52 (52, 61)	51 (39, 58)	49 (40, 59)	50 (40, 59)	0.412
Total body weight (kg)	Men	87.7 (80.3, 96.6)	85.3 (77.8, 95.4)	85.9 (78.0, 96.6)	86.4 (78.5, 96.3)	0.375
	Women	68.4 (61.1, 78.9)	70.2 (63.9, 79.1)	72.7 (64.1, 82.0)	70.1 (63.0, 79.8)	0.164
Body fat-free mass (kg)	Men	67.0 (62.7, 72.2)	66.9 (61.7, 71.0)	66.0 (60.4, 72.8)	66.7 (61.6, 72.2)	0.688
	Women	46.0 (43.8, 50.5)	47.0 (43.9, 50.5)	47.4 (44.2, 51.2)	46.9 (43.9, 50.7)	0.717
Body fat mass (kg)	Men	21.4 (16.4, 25.3)	19.4 (16.2, 24.5)	20.0 (16.4, 25.1)	20.1 (16.3, 25.1)	0.234
	Women	21.8 (17.1, 28.6)	23.4 (17.9, 30.8)	24.8 (18.5, 32.3)	23.0 (18.0, 30.5)	0.063
Height (cm)	Men	177 (173, 182)	179 (174, 183)	177 (173, 181)	178 (173, 182)	0.361
	Women	164 (159, 169)	165 (161, 169)	164 (160, 168)	164 (159, 169)	0.326
Body mass index (kg/m^2^)	Men	28.2 (25.8, 30.5)	27.3 (24.9, 30.1)	27.4 (25.0, 30.0)	27.7 (25.3, 30.2)	0.097
	Women	25.7 (22.6, 28.9)	25.8 (22.8, 30.6)	26.7 (23.8, 30.5)	26.0 (23.1, 30.1)	0.219
Waist circumference (cm)	Men	95.9 (88.5, 104)	93.4 (86.6, 103)	93.6 (87.0, 103)	94.0 (87.4, 103)	0.192
	Women	80.0 (73.0, 89.7)	80.0 (73.5, 92.0)	81.3 (76.0, 90.2)	81.0 (73.8, 90.5)	0.550
Waist-to-height ratio	Men	0.55 (0.49, 0.58)	0.53 (0.48, 0.58)	0.53 (0.50, 0.58)	0.54 (0.49, 0.58)	0.122
	Women	0.50 (0.44, 0.54)	0.49 (0.44, 0.56)	0.50 (0.46, 0.56)	0.50 (0.45, 0.55)	0.532
Systolic blood pressure (mmHg)	Men	132 (125, 142)	133 (123, 142)	135 (125, 145)	133 (124, 143)	0.583
	Women	116 (106, 127)	116 (108, 127)	119 (109, 131)	117 (108, 128)	0.333
Diastolic blood pressure (mmHg)	Men	78 (73, 85)	80 (75, 86)	81 (74, 88)	80 (74, 86)	0.243
	Women	74 (67, 79)	74 (68, 81)	74 (69, 81)	74 (68, 81)	0.236
Hypertension (%)	Men	53.9	44.2	50.3	50.5	0.228
	Women	38.9	30.8	33.1	34.3	0.358
Antihypertensive medication (%)	Men	37.2	23.7	21.9	27.6	**0.004**
	Women	32.1	22.3	26.2	29.9	0.201
Glycated hemoglobin (%)	Men	5.3 (4.9, 5.5)	5.3 (5.0, 5.7)	5.3 (5.1, 5.7)	5.3 (4.9, 5.6)	0.159
	Women	5.1 (4.7, 5.4)	5.2 (4.8, 5.5)	5.3 (4.9, 5.5)	5.2 (4.8, 5.5)	0.107
Type 2 diabetes mellitus (%)	Men	10.9	6.41	7.74	8.35	0.375
	Women	7.63	6.15	4.62	6.14	0.629
Hypoglycemic medication (%)	Men	6.41	3.85	1.94	4.07	0.146
	Women	3.05	0.00	0.00	1.02	**0.036**
Total cholesterol (mmol/l)	Men	5.20 (4.50, 6.00)	5.30 (4.70, 6.10)	5.50 (4.80, 6.20)	5.30 (4.70, 6.10)	**0.026**
	Women	5.50 (5.00, 6.20)	5.50 (4.60, 6.30)	5.40 (4.90, 6.20)	5.50 (4.90, 6.20)	0.941
LDL-cholesterol (mmol/l)	Men	3.34 (2.73, 3.86)	3.43 (2.86, 3.96)	3.59 (2.85, 4.06)	3.44 (2.83, 3.98)	**0.046**
	Women	3.33 (2.84, 3.77)	3.25 (2.63, 4.14)	3.42 (2.79, 4.00)	3.33 (2.76, 3.95)	0.485
HDL-cholesterol (mmol/l)	Men	1.25 (1.08, 1.45)	1.30 (1.11, 1.51)	1.33 (1.13, 1.52)	1.28 (1.11, 1.49)	0.053
	Women	1.59 (1.29, 1.87)	1.60 (1.38, 1.83)	1.61 (1.36, 1.87)	1.60 (1.35, 1.86)	0.802
Total cholesterol/HDL-C ratio	Men	4.18 (3.53, 4.83)	4.17 (3.37, 5.05)	4.29 (3.37, 4.96)	4.19 (3.41, 4.93)	0.957
	Women	3.37 (2.89, 4.29)	3.32 (2.73, 4.15)	3.51 (2.86, 4.17)	3.38 (2.86, 4.17)	0.569
Hypercholesterolemic (%)	Men	43.6	45.5	44.5	44.5	0.943
	Women	38.2	35.4	32.3	35.3	0.612
Lipid-lowering medication (%)	Men	14.1	6.41	5.16	8.57	**0.013**
	Women	9.16	1.54	3.08	4.60	**0.011**
Estimated glomerular filtration rate (mL/min/1.73 m^2^)	Men	90.2 (79.3, 103)	94.9 (81.9, 105)	94.6 (86.1, 107)	93.9 (81.9, 105)	**0.039**
	Women	93.5 (82.6, 104)	92.6 (79.5, 102)	92.4 (84.8, 105)	91.7 (82.6, 104)	0.650
Smoking (%)	Men					
Never		33.3	32.7	32.9	33.0	
Current		16.7	22.4	28.4	22.5	
Former		50.0	44.9	38.7	44.5	0.136
	Women					
Never		51.9	51.5	40.8	48.1	
Current		21.4	20.0	26.9	22.8	
Former		26.7	28.5	32.3	29.2	0.350

Data are expressed as median 25th and 75th percentile (continuous data) or percentage (categorical data)

A *p*-value *p* < 0.05 was considered as statistically significant and therefore highlighted in bold

*p-values are based on the chi-squared test (for cells with less than 10 individuals the *p*-values are based on the Fisher´s exact test) for categorical variables and the Kruskal–Wallis tests for continuous variables

### Reversion of the x-axis scale for serum S1P levels

While a previous study[27] of our research group, designed to define reference values with a sub-sample of 1339 healthy participants from the SHIP-TREND-0 cohort, showed that S1P concentrations were not associated with aging for men and women, the analyses of the whole population sample with all subjects with S1P measurements (*n* = 4194) showed that older age was related to lower values of S1P in both men and women (Supplementary Figure II), probably as a result of the presence of unhealthy participants. In line with that, the core objective of our study were the associations of lower values of S1P concentrations with parameters of cardiac geometry and systolic function, after adjustment for age and other covariates. Accordingly, all relations between S1P and heart variables were plotted with a reverse x-axis to permit a more intuitive analysis of the results.

### Associations of S1P values with structural parameters of LV

**Fig. 1:**
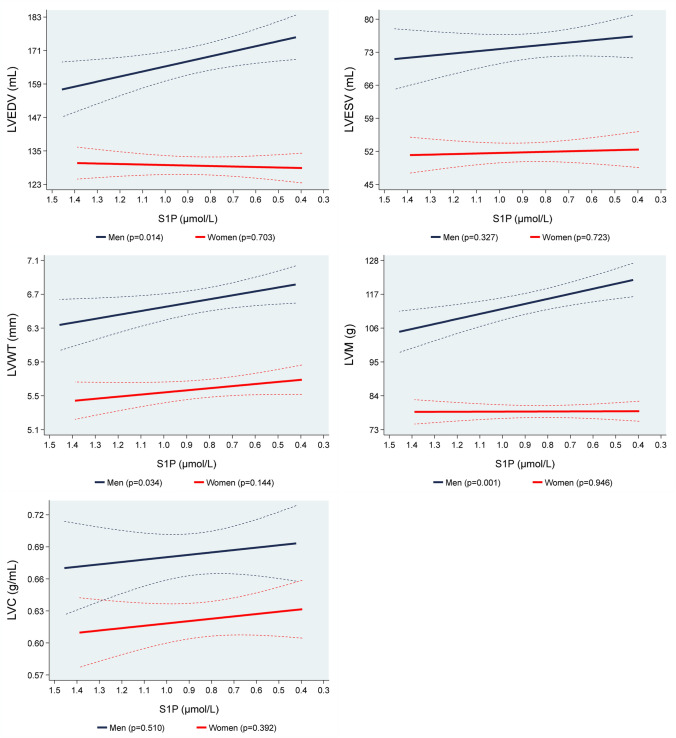
Adjusted* line (95% CI) showing the associations between sphingosine-1-phosphate (S1P) with mean magnetic resonance imaging determined left ventricular end-diastolic volume (LVEDV), left ventricular end-systolic volume (LVESV), left ventricular wall-thickness (LVWT), left ventricular mass (LVM) and left ventricular concentricity (LVC) stratified by sex (men = 457; women = 381). *Linear regression adjusted for age, body fat mass, body fat-free mass, height^2.7^, systolic blood pressure, use of antihypertensive medication, glycated hemoglobin, use of hypoglycemic medication, smoking status and eGFR. Data was weighted according to subjects that did not take part in the MRI examination

Figure 1 shows the associations of S1P concentrations with MRI determined LVEDV, LVWT, LVM and LVC. In multivariable adjusted regression analyses we observed statistically significant inverse associations of S1P concentrations with LVEDV, LVWT and LVM for men, while none of these associations was found for women. In detail, in men, a 1 µmol/L lower S1P concentration was associated with an 18.1 mL (95% confidence interval [CI] 3.66–32.6; *p* = 0.014) bigger LVEDV, a 0.46 mm (95% CI 0.04–0.89; *p* = 0.034) higher LVWT and a 16.3 g (95% CI 6.55–26.1; *p* = 0.001) greater LVM (Table 2, Supplemental Figure III). We could not show significant associations of S1P concentrations with LVESV and LVC for both sexes.

**Table 2:** Adjusted* ß-coefficient (95%-CI) of the associations of S1P with magnetic resonance imaging determined leftventricular and leftatrial cardiac geometry and function parameters stratified by sex (LV: men = 457; women = 381, LA: men = 421; women = 354)

Parameter	Men ß-coefficient (95% CI), *p*-value	Women ß-coefficient (95% CI), *p*-value
Left ventricular end-diastolic volume (mL)	− 18.1 (− 32.6 to − 3.66), ***p* = 0.014**	1.78 (− 7.34 to 10.9), *p* = 0.703
Left ventricular end-systolic volume (mL)	− 4.63 (− 13.9 to 4.63), *p* = 0.327	− 1.19 (− 7.77 to 5.40), *p* = 0.723
Left ventricular wall-thickness (mm)	− 0.46 (− 0.89 to − 0.04), ***p* = 0.034**	− 0.25 (− 0.58 to 0.09), *p* = 0.144
Left ventricular mass (g)	− 16.3 (− 26.1 to − 6.55), ***p* = 0.001**	− 0.21 (− 6.37 to 5.94), *p* = 0.946
Left ventricular concentricity (g/mL)	− 0.02 (− 0.09 to 0.04), *p* = 0.510	− 0.02 (− 0.07 to 0.03), *p* = 0.392
Left ventricular stroke volume (mL/beat)	− 13.3 (− 22.1 to − 4.49), ***p* = 0.003**	3.10 (− 4.03 to 10.2), *p* = 0.393
Left ventricular stroke work (cJ)	− 18.7 (− 30.9 to − 6.43), ***p* = 0.003**	5.10 (− 3.78 to 13.9), *p* = 0.260
Heart rate (bpm)	3.91 (− 2.20 to 10.0), *p* = 0.209	− 1.72 (− 8.52 to 5.10), *p* = 0.619
Left ventricular cardiac output (L/min)	− 0.62 (− 1.27 to 0.03), *p* = 0.061	0.08 (− 0.53 to 0.70), *p* = 0.800
Left ventricular ejection fraction (%)	− 1.91 (− 5.00 to 1.13), *p* = 0.218	1.14 (− 2.55 to 4.82), *p* = 0.544
Left atrial end-diastolic volume (mL)	− 12.6 (− 24.3 to − 1.03), ***p* = 0.033**	− 3.07 (− 11.6 to 5.41), *p* = 0.477
Left atrial end-systolic volume (mL)	− 7.42 (− 15.9 to 1.03), *p* = 0.085	2.19 (− 4.60 to 8.95), *p* = 0.524
Left atrial stroke volume (mL/beat)	− 4.03 (− 9.85 to 1.80), *p* = 0.175	− 4.29 (− 12.6 to 4.02), *p* = 0.311
Left atrial cardiac output (L/min)	− 0.10 (0.59 to 0.40), *p* = 0.706	− 0.39 (− 1.05 to 0.27), *p* = 0.242
Left atrial ejection fraction (%)	1.08 (− 3.12 to 5.28), *p* = 0.613	− 0.34 (− 4.82 to 4.13), *p* = 0.880

A *p*-value *p* < 0.05 was considered as statistically significant and therefore highlighted in bold

*Linear regression adjusted for age, body fat mass, body fat-free mass, height^2.7^, systolic blood pressure, use of antihypertensive medication, glycated hemoglobin, use of hypoglycemic medication, smoking status and eGFR. Data was weighted according to subjects that did not take part in the MRI examination

In sensitivity analyses, in men, we found that a 1 µmol/L lower S1P concentration was associated with a 21.6 g (95% CI 6.46–36.7; *p* = 0.005) higher LVM in hypertensive individuals, but there was no association in normotensive subjects (*p* = 0.306). There were no associations of S1P levels with LVM for both hypertensive and normotensive women. Likewise, in men, we observed that a 1 µmol/L lower S1P concentration was associated with a 27.9 g (95% CI 9.76–46.0; *p* = 0.003) and a 17.3 g (95% CI 1.73–32.8; *p* = 0.030) greater LVM in current smokers and formers smokers, respectively, but there was no association in never smokers (*p* = 0.270). There were no associations of S1P levels with LVM regarding smoking status in women. We also performed sensitive analyses to evaluate the associations of S1P with LVM stratified by menopausal status. Both premenopausal and postmenopausal women had no significant associations. Noteworthy, the analyses groups became too small after stratification, which might have influenced the results.

### Associations of S1P values with systolic parameters of LV

**Fig. 2:**
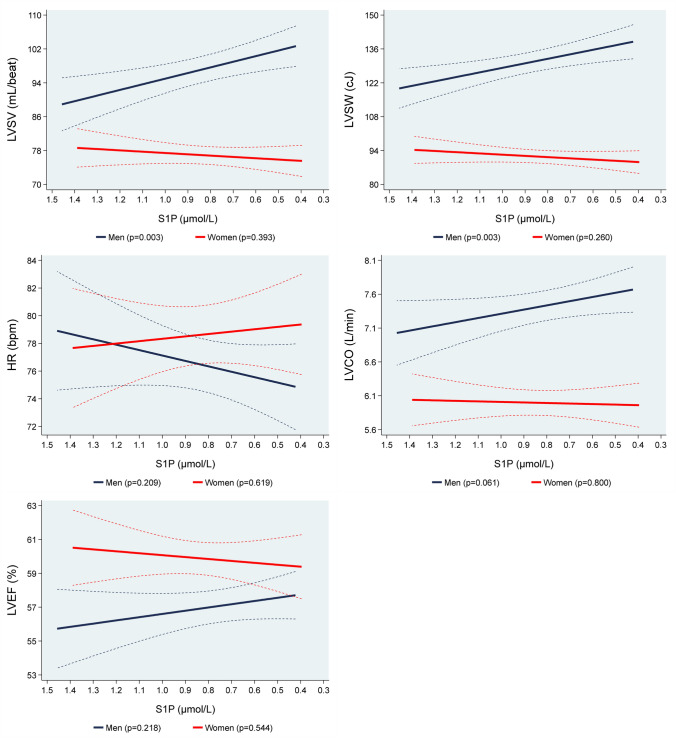
Adjusted* line (95% CI) showing the associations between sphingosine-1-phosphate (S1P) with mean magnetic resonance imaging determined left ventricular stroke volume (LVSV), left ventricular stroke work (LVSW), heart rate (HR), left ventricular cardiac output (LVCO) and left ventricular ejection fraction (LVEF) stratified by sex (men = 457; women = 381). *Linear regression adjusted for age, body fat mass, body fat-free mass, height^2.7^, systolic blood pressure, use of antihypertensive medication, glycated hemoglobin, use of hypoglycemic medication, smoking status and eGFR. Data was weighted according to subjects that did not take part in the MRI examination

While we found inverse associations of S1P concentrations with LVSV and LVSW in men, we did not observe associations of these parameters in women. Specifically, in men, a 1 µmol/L lower S1P concentration was associated with a 13.3 mL/beat (95% CI 4.49–22.1; *p* = 0.003) higher LVSV and an 18.7 cJ (95% CI 6.43–30.9; *p* = 0.003) higher LVSW (Table 2, Supplemental Figure IV). There were no associations of S1P concentrations with HR, LVCO and LVEF for both sexes (Fig. 2).

### Associations of S1P values with structural and systolic parameters of LA

**Fig. 3:**
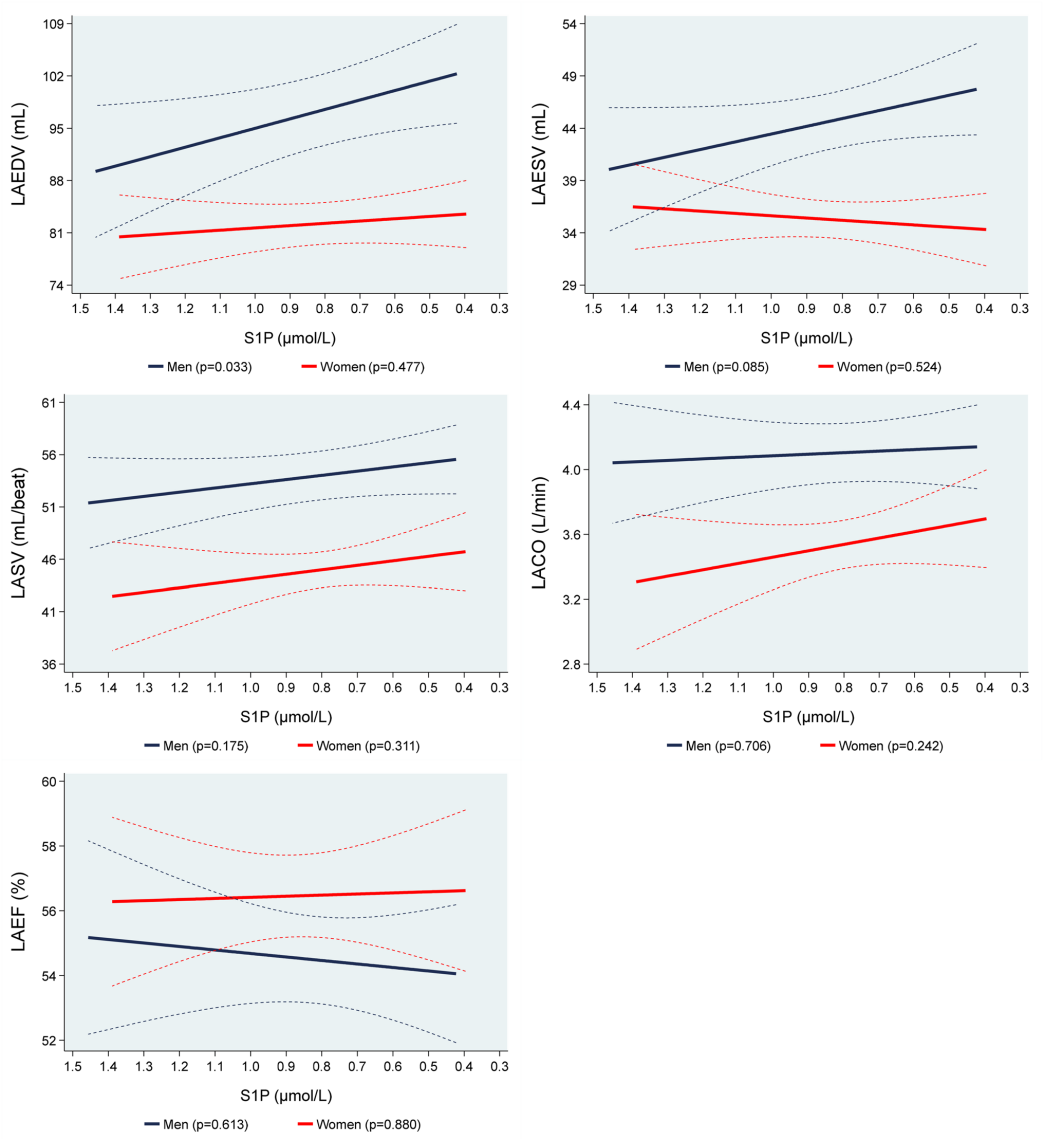
Adjusted* line (95% CI) showing the associations between sphingosine-1-phosphate (S1P) with mean magnetic resonance imaging left atrial end-diastolic volume (LAEDV), left atrial end-systolic volume (LAESV), left atrial stroke volume (LASV), left atrial cardiac output (LACO) and left atrial ejection fraction (LAEF) stratified by sex (men = 421; women = 354). *Linear regression adjusted for age, body fat mass, body fat-free mass, height^2.7^, systolic blood pressure, use of antihypertensive medication, glycated hemoglobin, use of hypoglycemic medication, smoking status and eGFR. Data was weighted according to subjects that did not take part in the MRI examination

After multivariable regression analyses we found a statistically significant inverse association of S1P concentrations with LAEDV in men, but not in women. In detail, in men, a 1 µmol/L lower S1P concentration was associated with a 12.6 mL (1.03–24.3; *p* = 0.033) bigger LAEDV (Table 2, Supplemental Figure V). There were no associations of S1P concentrations with LAESV, LASV, LACO and LAEF for both men and women (Fig. 3).

## Discussion

**Fig. 4:**
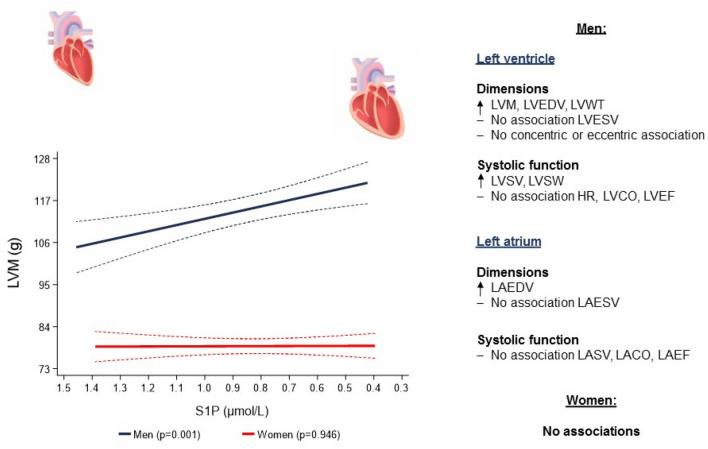
Associations of sphingosine-1-phosphate levels with left atrial and left ventricular geometry and systolic function parameters

In our community-based sample, we found inverse associations of S1P concentrations with structural and systolic function LV and LA parameters. Importantly, these associations were sex-specific and detectable only in men, but not in women. Specifically, we found that, lower S1P concentrations were associated with larger LV and LA volumes, thicker LVWT and higher LVM, LVSV and LVSW (Fig. 4).

### In the context of the published literature

While there were some studies [34, 35] that described that higher S1P levels were related with hazardous outcomes, several other analyses [11, 12, 14, 15, 36], in agreement with our findings, showed the opposite, i.e. that lower S1P concentrations were associated with pathophysiologic clinical conditions.

A previous animal study [36], with pressure-overloaded cardiomyocytes, showed that the use of S1P could inhibit cardiomyocyte autophagy, thus preventing cardiomyocyte hypertrophy and consequently, protecting the cardiac function, mainly through the activation of the S1PR1.

A clinical study [14] among 74 patients (68% men, mean age of 73 years) with ischemic heart disease showed that lower S1P levels were related to a lower LVEF, as assessed by echocardiography, and a higher severity of heart failure determined by NYHA class. Our findings did not confirm the associations of S1P levels with LVEF or LVCO in both men and women. In this context, however, it must be considered that our sample excluded participants with compromised LV function, which indicate that our results reflect a subclinical stage. Moreover, we used a population-based study with a higher number of participants, a broad age range, sex-specific analysis and the use of MRI to determine the cardiac parameters which is considered to be a more accurate method as compared to echocardiography [37, 38].

### Potential mechanisms for the observed associations

The associations of lower S1P levels with cardiac geometry and systolic function parameters might be the result of shared multiple risk factors and comorbidities, such as older age, obesity, hypertension, type 2 diabetes and smoking which can explain these associations as parallel relations rather than direct ones. On the other hand, we have adjusted for many risk factors in our multivariable regression models with no significant modification of our results, which might advocate an independent association of lower S1P levels with these cardiac parameters. Our findings were only significant in men, which suggest that sex hormones might have an important influence, particularly estrogen, which is known for cardioprotective effects. Interestingly, ovariectomized rats experience a decrease of expression of sphingosine kinases 1 and 2, the key enzymes involved in S1P synthesis, alongside with decrease S1P concentrations in aortic tissue [39]. This phenotype was rescued by estradiol valerate treatment. On the other hand, in our sensitive analyses, both premenopausal and postmenopausal women had no significant associations of S1P with LVM.

The role of S1P in heart (patho) physiology is complex. Synthesis of S1P involves endothelial cells, thrombocytes, erythrocytes and other cells, being transported by various carriers including albumin, LDL- and HDL-cholesterol [40]. Activation of the S1P receptor 3 promotes cardiac fibroblast proliferation, but decreases collagen secretion [41, 42]. S1P inhibits proliferation of several muscular cell types via S1P receptor 2 activation and induces cell differentiation, e.g. to cardiomyocytes from of human mesenchymal stem cells [43–45]. Acute administration of the S1PR1, 3–5 agonist fingolimod (FTY720) induces bradycardia [46], but chronic administration in mouse models of heart transplantation or pressure overload protects against cardiac fibrosis, possibly by functional antagonism of S1PR1 in immune cells [47, 48]. Moreover, S1P plays a substantial role in regulation of blood pressure and vascular tone [49, 50]. S1P leads to a strong production of the endothelial nitric oxide (NO) synthase (eNOS)-derived NO in a similar extent like the vascular endothelial growth factor and bradykinin [50]. Mechanistically, S1P-depended activation of S1PR1, the predominant receptor-subtype in endothelial cells, leads through stimulation of the PI3K/Akt/eNOS pathway to endothelial release of NO[51] and a subsequent vasodilatation. Accordingly, lower S1P concentrations might result in decreased NO-liberation and facilitated endothelial dysfunction with a consequent increase in vascular tone. This would result in a raise of the cardiac afterload and subsequent boost of the LVSW. The concomitant increase in the myocardial wall tension would lead to an expand in the LVWT and LVEDV, with an accompanying enlargement of the LVSV and LAEDV, resulting in an enhanced LVM. Interestingly, our findings in sensitivity analyses, that S1P levels were associated with LVM in hypertensive and smoker men might suggest that the associations of low S1P levels could be accentuated in clinical conditions related with endothelial dysfunction.

## Study limitations

Our study has some limitations that are needed to be mentioned. Our study only consisted of Caucasians, therefore, extrapolation to other ethnicities is not appropriate. Since we have a non-random subsample, we cannot exclude a selection bias. Although our cardiac MRI dataset provided detailed information on structural parameters and on parameters of systolic function, we have no data regarding diastolic function. Additionally, while we have adjusted as best as possible for confounding factors, causal assumptions have still to be done with caution due to the cross-sectional study design and the possibility of residual confounding. Future longitudinal studies and replication might help to elucidate these associations.

Notwithstanding, our study has also some important strengths including the large sample size, the standardized assessment of MRI with detailed measurement of cardiac geometry and function and the possibility to adjust for multiple metabolic risk factors like body fat mass, body fat-free mass, glycated hemoglobin, that were available in our study.

## Conclusions

Our results indicate that lower levels of S1P were associated with parameters reflecting cardiac geometry and systolic function in men. Specifically, in this population-based sample, lower levels of S1P were associated with higher LV wall thickness and mass, larger LV and LA chamber sizes and greater stroke volume and work of the LV in men, but not in women. Further experimental and clinical studies investigating the S1P signaling pathway in cardiovascular pathology should in particular be vigilant on sex differences and impact of sex hormones.

## Perspectives

### Clinical competencies

In our study we demonstrated that lower S1P concentrations were associated with larger chamber sizes and higher wall-thickness, stroke volume, stroke work and mass of the left heart in men. We believe these results indicate that S1P might be associated with cardiac geometry and systolic function parameters in men.

### Translational outlook

Future longitudinal studies are necessary to substantiate the associations of S1P and heart geometry, as well as its sexual dimorphism that we found. Since most experimental studies investigating S1P are only performed with male animals [7], future studies should include male and female individuals, that would be exposed to conditions leading to modifications of cardiac shape, size, structure and function. A more mechanistic design, such as genetic receptor knockout or use of S1PR- agonist/antagonist substances might clarify causal pathways and possible pharmacological interventions.

## Data Availability

The datasets generated during and/or analyzed during the current study are not publicly available due to data protection aspects but are available in an anonymized form from the corresponding author on reasonable request.

## Abbreviations

CKD-EPI: Chronic kidney disease epidemiology collaboration
CV: Cardiovascular
CVDs: Cardiovascular diseases
eGFR: Estimated glomerular filtration rate
HR: Heart rate
LA: Left atrial
LACO: Left atrial cardiac output
LAEDV: Left atrial end-diastolic volume
LAEF: Left atrial ejection fraction
LAESV: Left atrial end-systolic volume
LASV: Left atrial stroke volume
LV: Left ventricular
LVC: Left ventricular concentricity
LVCO: Left ventricular cardiac output
LVEDV: Left ventricular end-diastolic volume
LVEF: Left ventricular ejection fraction
LVESV: Left ventricular end-systolic volume
LVM: Left ventricular mass
LVSV: Left ventricular stroke volume
LVSW: Left ventricular stroke work
LVWT: Left ventricular wall thickness
MRI: Magnetic resonance imaging
SHIP: Study of Health in Pomerania
S1P: Sphingosine-1-phosphate
S1PR: Sphingosine-1-phosphate-receptor
